# Supplementary material for: Inverse Relationship between Mean Corpuscular Volume and T-Score in Chronic Dialysis Patients
Source: Medicina (Kaunas). 2022 Mar 30;58(4):497. doi: 10.3390/medicina58040497 (PMC9032450; doi:10.3390/medicina58040497)
Supplement: Supplementary file 1 [file medicina-58-00497-s001.zip › medicina-1625841-supplementary.pdf]

Supplementary Table S1. Correlation between baseline clinical characteristics with MCV or T-score

Clinical characteristics (n=123)	*p-value* of correlation with MCV	Pearson correlation index	*p-value* of correlation with T-score	Pearson correlation index
**Age**	**0.01**	**0.23**	**< 0.001**	**-0.32**
**Gender**	0.8		0.23	
Male				
Female				
**BMI**	0.4		**0.004**	**0.26**
**Mean duration of dialysis** (months)	0.9		0.6	
**Types of dialysis**	0.2		0.76	
Hemodialysis				
Peritoneal dialysis				
**Underlying comorbidities**				
Hypertensions	0.1		0.9	
Diabetes mellitus	0.1		0.9	
Dyslipidemia	0.8		**0.09**	
Gastrointestinal tract ulcer history	0.5		**0.10**	
**Smoking history**	0.4		0.65	
Quitted				
Current smoker				
**Laboratory parameters**				
Hemoglobin, g/dl	**< 0.001**	**0.2**	0.6	
RBC $\times 10^6/\mu L$	**< 0.001**	**-0.65**	0.12	
MCV, fl	NA		**0.003**	**-0.27**
RDW, %	**< 0.001**	**-0.58**	0.8	
WBC count $\times 10^3/L$	**0.08**	**-0.16**	0.9	
Albumin, g/dl	0.2		0.2	
Phosphate, mg/dl	**0.008**	**-0.24**	**0.002**	**0.28**
Ca, mg/dl	0.3		0.47	
Corrected Ca, mg/dl	0.7		0.95	
Iron, ug/dL	**0.06**	**0.17**	0.5	
Ferritin, ng/mL	**0.035**	**0.2**	0.16	
Cholesterol	**0.02**	**0.21**	0.14	
GOT, U/L	0.2		0.15	
GPT, U/L	0.7		0.4	
**Parathyroid hormone level** (pg/mL)	0.8		**0.037**	**-0.19**

Abbreviations: BMI, body mass index; Ca, calcium; GOT, glutamate oxaloacetate transaminase; GPT, glutamate pyruvic transaminase; MCV, mean corpuscular volume; RDW, red cell distribution width; SD, standard deviation.

Supplementary Table S2. Multivariate linear regression of potential risk factors affecting T-score among Taiwanese hemodialysis patients.

**HD patients (n = 90)**	Variables	β	*p-value*
Model 1 (MCV was set as the dependent variable)	**T-score**	**-0.97**	**0.004**
	Age	0.12	0.06
	Phosphate	-0.4	0.3
	**RDW**	**-1.6**	**< 0.001**
	Hb	1.06	0.1
	WBC	-0.6	0.08
	Iron	0.01	0.7
	Ferritin	0.004	0.1
	Cholesterol	0.002	0.9
Model 2 (T-score was set as the dependent variable)	**MCV**	**-0.036**	**0.04**
	**Age**	**-0.036**	**0.01**
	Phosphate	0.07	0.4
	**BMI**	**0.07**	**0.02**
	**Parathyroid hormone**	**-0.001**	**0.048**
	Dyslipidemia (No)	-0.3	0.6
	GI tract ulcer history (No)	0.7	0.2
Model 3 (T-score was set as the dependent variable)	**MCV**	**-0.04**	**0.036**
	**Age**	**-0.03**	**0.02**
	WBC	-0.009	0.9
	Albumin	0.17	0.7
	GOT	-0.013	0.6
	GPT	0.004	0.9

Model 1: MCV was set as the dependent variable and adjusted for T-score, age, serum phosphate level, RDW, Hb, WBC, iron, ferritin, and cholesterol.

Model 2: T-score was set as the dependent variable and adjusted for MCV, age, serum phosphate level, BMI, parathyroid hormone level, dyslipidemia, and gastrointestinal tract ulcer history.

Model 3: T-score was set as the dependent variable and adjusted for MCV, age, WBC, albumin, GOT, and GPT.

Abbreviations: BMI, body mass index; GOT, glutamate oxaloacetate transaminase; GPT, glutamate pyruvic transaminase; Hb, hemoglobin; MCV, mean corpuscular volume; RDW, red blood distribution width; WBC, white blood cell.

Supplementary Table S3. Multivariate linear regression of potential risk factors affecting T-score among Taiwanese peritoneal dialysis patients.

**PD patients (n = 33)**	Variables	β	*p-value*
Model 1 (MCV was set as the dependent variable)	T-score	-1.1	0.5
	Age	0.03	0.8
	Phosphate	-0.4	0.7
	RDW	-1.4	0.2
	Hb	-0.08	0.1
	WBC	0.03	1.0
	Iron	0.04	0.3
	Ferritin	0.001	0.7
	Cholesterol	0.1	0.06
Model 2 (T-score was set as the dependent variable)	MCV	0.01	0.7
	**Age**	**-0.04**	**0.01**
	Phosphate	-0.01	0.9
	BMI	0.07	0.2
	Parathyroid hormone	-0.001	0.05
	Dyslipidemia (No)	-0.6	0.6
	GI tract ulcer history (No)	-0.5	0.7
Model 3 (T-score was set as the dependent variable)	MCV	0.006	0.8
	**Age**	**-0.03**	**0.04**
	WBC	-0.1	0.13
	Albumin	0.37	0.4
	GOT	-0.06	0.14
	GPT	0.006	0.8

Model 1: MCV was set as the dependent variable and adjusted for T-score, age, serum phosphate level, RDW, Hb, WBC, iron, ferritin, and cholesterol.

Model 2: T-score was set as the dependent variable and adjusted for MCV, age, serum phosphate level, BMI, parathyroid hormone level, dyslipidemia, and gastrointestinal tract ulcer history.

Model 3: T-score was set as the dependent variable and adjusted for MCV, age, WBC, albumin, GOT, and GPT.

Abbreviations: BMI, body mass index; GOT, glutamate oxaloacetate transaminase; GPT, glutamate pyruvic transaminase; Hb, hemoglobin; MCV, mean corpuscular volume; RDW, red blood distribution width; WBC, white blood cell.
